# Development of Nomograms for Predicting Lymph Node Metastasis and Distant Metastasis in Newly Diagnosed T1-2 Non-Small Cell Lung Cancer: A Population-Based Analysis

**DOI:** 10.3389/fonc.2021.683282

**Published:** 2021-09-08

**Authors:** Yiming Qi, Shuangshuang Wu, Linghui Tao, Yunfu Shi, Wenjuan Yang, Lina Zhou, Bo Zhang, Jing Li

**Affiliations:** ^1^Department of Oncology, Tongde Hospital of Zhejiang Province, Hangzhou, China; ^2^Department of Geriatrics, Tongde Hospital of Zhejiang Province, Hangzhou, China; ^3^The Second Clinical Medical College of Zhejiang Chinese Medical University, Hangzhou, China; ^4^Integrated Chinese and Western Medicine, Cancer Hospital of the University of Chinese Academy of Sciences (Zhejiang Cancer Hospital), Institute of Cancer and Basic Medicine (IBMC), Chinese Academy of Sciences, Hangzhou, China; ^5^Cancer Institute of Integrative Medicine, Tongde Hospital of Zhejiang Province, Hangzhou, China

**Keywords:** SEER database, T1-2 non-small cell lung cancer, lymph node metastasis, distant metastasis, nomogram

## Abstract

**Background:**

For different lymph node metastasis (LNM) and distant metastasis (DM), the diagnosis, treatment and prognosis of T1-2 non-small cell lung cancer (NSCLC) are different. It is essential to figure out the risk factors and establish prediction models related to LNM and DM.

**Methods:**

Based on the surveillance, epidemiology, and end results (SEER) database from 1973 to 2015, a total of 43,156 eligible T1-2 NSCLC patients were enrolled in the retrospective study. Logistic regression analysis was used to determine the risk factors of LNM and DM. Risk factors were applied to construct the nomograms of LNM and DM. The predictive nomograms were discriminated against and evaluated by Concordance index (C-index) and calibration plots, respectively. Decision curve analysis (DCAs) was accepted to measure the clinical application of the nomogram. Cumulative incidence function (CIF) was performed further to detect the prognostic role of LNM and DM in NSCLC-specific death (NCSD).

**Results:**

Eight factors (age at diagnosis, race, sex, histology, T-stage, marital status, tumor size, and grade) were significant in predicting LNM and nine factors (race, sex, histology, T-stage, N-stage, marital status, tumor size, grade, and laterality) were important in predicting DM(all, P< 0.05). The calibration curves displayed that the prediction nomograms were effective and discriminative, of which the C-index were 0.723 and 0.808. The DCAs and clinical impact curves exhibited that the prediction nomograms were clinically effective.

**Conclusions:**

The newly constructed nomograms can objectively and accurately predict LNM and DM in patients suffering from T1-2 NSCLC, which may help clinicians make individual clinical decisions before clinical management.

## Introduction

Lung cancer is one of the most common malignant tumors. According to statistics, another 228,820 lung cancer cases were discovered in the United States in 2020, while 135,720 patients died of the disease. The incidence rate and mortality rate of lung cancer were the highest in all malignant tumors (1). Lung cancer can be divided into small cell lung cancer (SCLC) and non-small cell lung cancer (NSCLC) based on the pathological classification, in which NSCLC accounts for 85% of the newly diagnosed lung cancer (2). According to the American Joint Commission on Cancer (AJCC) TNM 7th edition staging system, T1-2 refers to that the maximum diameter of the primary tumor is ≤ 7cm; the chest lesions are limited; the chest wall, transverse septum, mediastinal pleura, pericardium, trachea, and esophagus were not involved; no satellite nodules were found in the lung, either. In terms of patients with newly diagnosed pulmonary space occupying lesions, if they are suspected of malignant tumor, percutaneous biopsy, bronchoscopy, sputum cytology, and other methods will be adopted to clarify the pathology before the operation. High-resolution CT (HRCT) can make the clinical T-stage. Most of T1-2 NSCLC patients had no LNM and DM at the initial diagnosis, but some T1-2 NSCLC patients had LNM and/or DM. For N and M staging, further examination is quite necessary. The standard examinations are as follows: PET-CT, lymph node biopsy, mediastinoscopy, surgery. Although there are clinical guidelines for further assessment and treatment, many doctors still cannot fully understand and remember the contents of the guidelines, or cannot keep up with the disciplines’ progress, so they often make plans on the basis of local experience and personal experience. For example, for the clinical suspected 2R/2L, 4R/4L, and 10R/10L regional lymph node metastasis, the guidelines recommend esophageal ultrasound-guided biopsy. Still in clinical practices, some thoracic surgeons did not carry out this evaluation before the operation, while performed lymph node biopsy or dissection according to experience. If LNM and DM can be predicted accurately from the outset, the examination and treatment can be more targeted, diagnosis time may be shorter, the unnecessary examination can be rolled out, and patients’ economic burden can be reduced as well. Besides, as for whether there are lymph nodes and distant metastasis or not, the results of the prognosis of T1-2 NSCLC are entirely different. If we can predict LNM and DM, we can also more accurately judge the prognosis.

Nomogram, a graphical and straightforward prediction tool, can be used to numerically calculate the risk probability of clinical events for individual patients (3, 4). For many malignant tumors, the better predictive ability of nomogram has been confirmed, compared with the widely used TNM staging system (5, 6). However, up to now, it is still impossible to obtain an accurate nomogram for the prediction of LNM and DM in T1-2 lung cancer patients. Therefore, we aimed to evaluate T1-2 NSCLC using the LNM and DM nomograms in the SEER database.

## Methods

### Patient Enrollment and Characteristics

**Figure 1:**
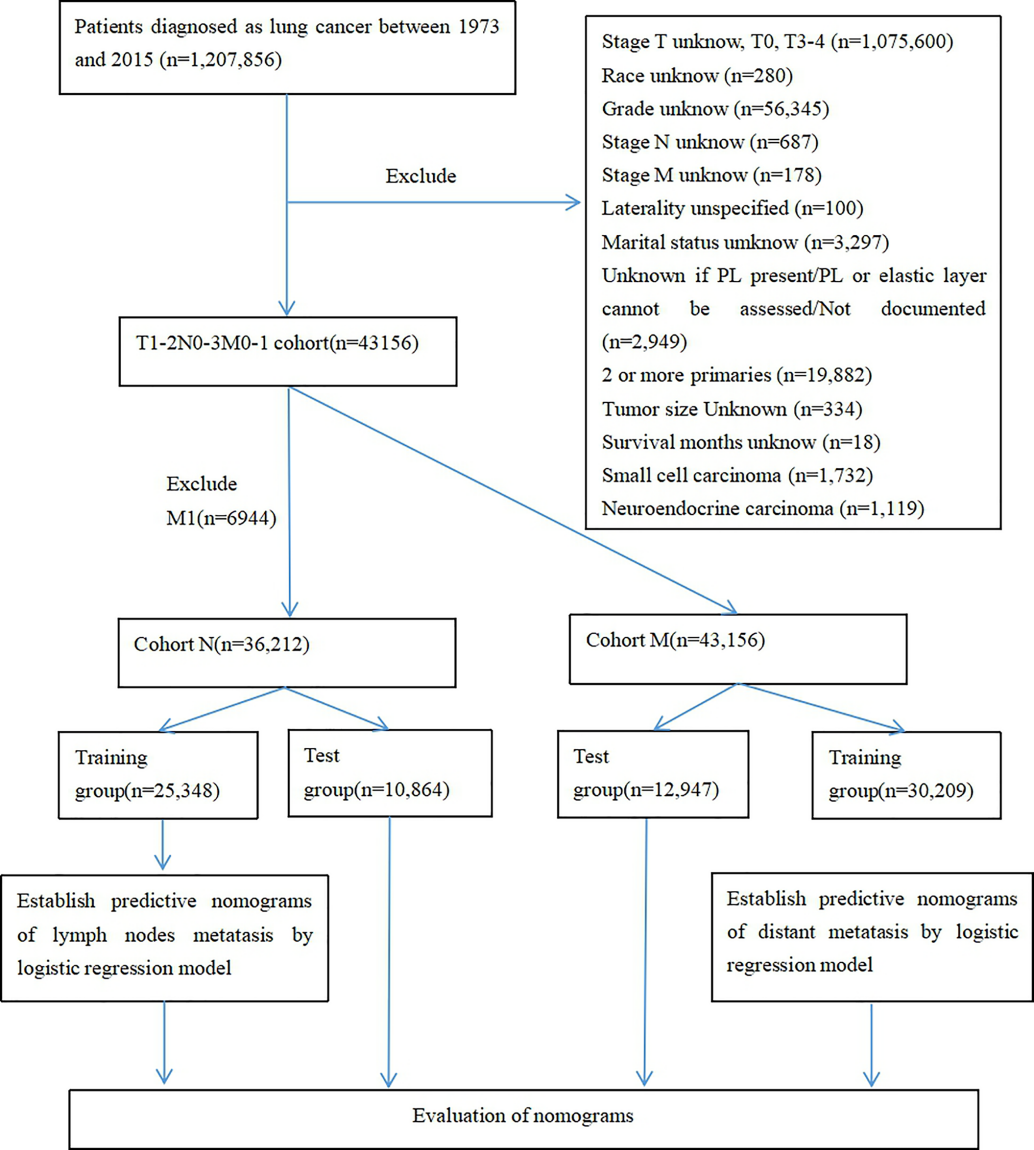
Case screening flow chart.

**Figure 2:**
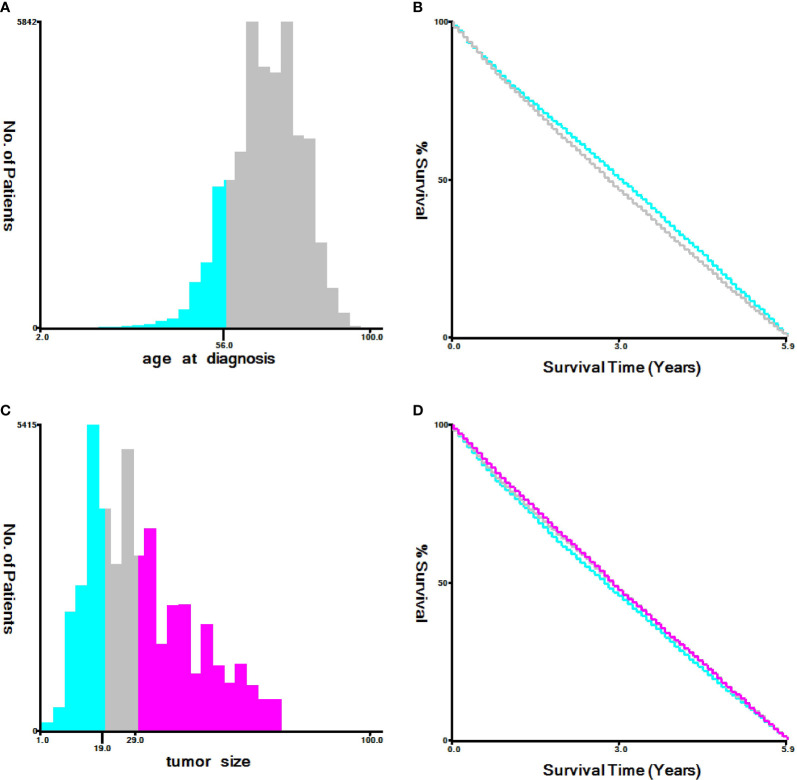
Identification of optimal cut-off values of age **(A, B)** and tumor size **(C, D)** *via* X-tile software analysis. Optimal cut-off values of age were identified as 56 based on NCSD. Optimal cut-off values of tumor size were determined as 19 mm and 29 mm based on NCSD.

We used seerstat8.3.6 software to extract data from the SEER database(http://seer.cancer.gov/seerstat/), and the authoritative cancer statistics database of US cover 34.6% of US population up to now. Within the SEER database, we enrolled 43,156 patients who were diagnosed with primary T1-2 non-small cell lung between 1973 and 2015. Lung cancer cases were screened according to the following factors: year of diagnosis, sex, age of diagnosis, race recode, marital status at diagnosis, laterality, ICD-O-3 Hist/behav,malignant, grade, CS tumor size (2004+), Derived AJCC TNM(7th), RX Summ–Scope Reg LN Sur(2003+), SEER cause of death classification, Vital status recode, survival months, and other SEER cause of death classification. The flowchart of case selection is illustrated in **Figure 1**. The optimal cut-off values for age and tumor size were assessed by X-tile software (Yale University, New Haven, Connecticut, USA) **(** **Figure 2** **)**. Patients in cohort N and cohort M were divided into the training group and test group in the ratio of 7:3, randomly.

### Variable Declaration

The histology variable was classified as “Adenocarcinomas,” “Squamous cell carcinoma,” “Large cell carcinoma” or “Other”. The cause-specific death was classified as “alive,” “dead due to cancer” or “dead due to other cause”. Meanwhile, “stage M1a” and “stage M1b” were classified as “stage M1”.

### Nomograms Construction

Univariate and multivariate logistic regression analyses were performed to identify risk factors for LNM and DM cases. The factors screened out by multiple logistic regression models (P < 0.05) were applied to construct the nomograms. C-index and calibration plots conducted by a bootstrapping method with 1000 resamples were used to validate the nomograms in the discriminatory power. The DCAs were plotted to validate the nomograms in clinical application value. Based on the DCAs, clinical impact curves were chosen to show the significant value of the nomograms. In addition, the CIF was carried out further to determine the prognostic role of LNM and DM in NSCLC-specific death (NCSD). All models and images were conducted by R software (version 3.5.1) with various packages, including foreign, rms, nom1, rmda, tibble, survival, cmprsk, and stdca (https://rstudio.com/products/rpackages/).

### Statistical Analysis

The optimal cut-off values for age and tumor size were assessed by the X-tile software Kaplan Meier curve. The baseline of patients between the training group and the test group was tested through Chi-square tests. The general situation of patients was summarized by Spss23.0. The difference was statistically significant if P < 0.05. Other data analyses were carried out through the corresponding functions of R software.

## Results

### Patients and Characteristics

After strict screening, 43,156 patients diagnosed with T1-2 NSCLC during 2010-2015 were finally included in this study from the SEER database. There were cohort N (T1-2N0-2M0 stage NSCLC, n = 36,212) and cohort M (T1-2 NSCLC, n = 43,156). The patients in cohort N were divided into training group (n = 25,348) and test group (n = 10,864). The patients in cohort M were divide into training group (n =30,209) and test group (n = 12,947). Totally, 9,439 of 36,212 patients (26.07%) occurred LNM in cohort N, and 6,944 of 43,156 patients (16.09%) occurred DM in cohort M. Patients’ characteristics were listed in **Tables 1**, **2**.

**Table 1:** Characteristics of patients in cohort N.

Characteristic	Nt (n=36212)	Training group (n = 25348)		Test group (n = 10864)		p
		Ne (%)	Nne (%)	Ne (%)	Nne (%)	
		N=6582	N=18766	N=2857	N=8007	
Age						0.405
≤56	4535 (12.52)	1012 (15.38)	2187 (11.65)	395 (13.83)	941 (11.75)	
>56	31677 (87.48)	5570 (84.62)	16579 (88.35)	2462 (86.17)	7066 (88.25)	
Race						<0.001
White	30107 (83.14)	5384 (81.80)	15750 (83.93)	2309 (80.82)	6664 (83.23)	
Black	3433 (9.48)	693 (10.53)	1626 (8.66)	345 (12.08)	769 (9.60)	
American Indian /Alaska Native	175 (0.48)	40 (0.61)	99 (0.53)	8 (0.28)	28 (0.35)	
Asian or Pacific Islander	2497 (6.90)	465 (7.06)	1291 (6.88)	195 (6.83)	546 (6.82)	
Sex						0.681
Male	17054 (47.09)	3458 (52.54)	8498 (45.28)	1484 (51.94)	3614 (45.14)	
Female	19158 (52.91)	3124 (47.46)	10268 (54.72)	1373 (48.06)	4393 (54.86)	
Histology						0.453
Adenocarcinomas	21240 (58.65)	3775 (57.35)	11069 (58.98)	1634 (57.19)	4762 (59.47)	
Squamous cell carcinoma	11283 (31.16)	2362 (35.89)	5531 (29.47)	1028 (35.98)	2362 (29.50)	
Large cell carcinoma	278 (0.77)	75 (1.14)	113 (0.60)	28 (0.98)	62 (0.77)	
Others	3411 (9.42)	370 (5.62)	2053 (10.94)	167 (5.85)	821 (10.25)	
T-stage						0.310
T1a	10967 (30.29)	937 (14.24)	6767 (36.06)	391 (13.69)	2872 (35.87)	
T1b	7401 (20.44)	1041 (15.82)	4074 (21.71)	505 (17.68)	1781 (22.24)	
T2a	13930 (38.47)	3212 (48.80)	6561 (34.96)	1387 (48.55)	2770 (34.59)	
T2b	3914 (10.81)	1392 (21.15)	1364 (7.27)	574 (20.09)	584 (7.29)	
Marital status						0.896
Married	20070 (55.42)	3719 (56.50)	10334 (55.07)	1595 (55.83)	4422 (55.23)	
Single	5145 (14.21)	937 (14.24)	2661 (14.18)	419 (14.67)	1128 (14.09)	
Divorced	4676 (12.91)	881 (13.38)	2410 (12.84)	378 (13.23)	1007 (12.58)	
Widowed	6321 (17.46)	1045 (15.88)	3361 (17.91)	465 (16.28)	1450 (18.11)	
Tumor size						0.437
1-19	10642 (29.39)	884 (13.43)	6587 (35.10)	384 (13.44)	2787 (34.81)	
20-29	10271 (28.36)	1467 (22.29)	5672 (30.22)	668 (23.38)	2464 (30.77)	
>29	15299 (42.25)	4231 (64.28)	6507 (34.67)	1805 (63.18)	2756 (34.42)	
Grade						0.496
Well differentiated; Grade I	6754 (18.65)	479 (7.28)	4299 (22.91)	209 (7.32)	1767 (22.07)	
Moderately differentiated; Grade II	16178 (44.68)	2754 (41.84)	8560 (45.61)	1163 (40.71)	3701 (46.22)	
Poorly differentiated; Grade III	12853 (35.49)	3241 (49.24)	5717 (30.46)	1439 (50.37)	2456 (30.67)	
Undifferentiated; Grade IV	427 (1.18)	108 (1.64)	190 (1.01)	46 (1.61)	83 (1.04)	
Laterality						0.567
Left	14883 (41.10)	2764 (41.99)	7679 (40.92)	1174 (41.09)	3266 (40.79)	
Right	21329 (58.90)	3818 (58.01)	11087 (59.08)	1683 (58.91)	4741 (59.21)	
Survival statue						0.452
Dead	11290 (31.18)	3271 (49.70)	4601 (24.52)	1384 (48.44)	2034 (25.40)	
Alive	24922 (68.82)	3311 (50.30)	14165 (75.48)	1473 (51.56)	5973 (74.60)	

P-value indicates the baseline comparison between training group and test group.

**Table 2:** Characteristic of patients in cohort M.

Characteristic	Mt (%)N=43156	Training group N = 30209		Test group N = 12947		p
		Me (%)	Mne (%)	Me (%)	Mne (%)	
		N=4837	N=25372	N=2107	N=10840	
Age						0.416
≤56	5615 (13.01)	765 (15.82)	3192 (12.58)	315 (14.95)	1343 (12.39)	
>56	37541 (86.99)	4072 (84.18)	22180 (87.42)	1792 (85.05)	9497 (87.61)	
Race						0.633
White	35597 (82.48)	3803 (78.62)	21118 (82.23)	1687 (80.07)	8989 (82.92)	
Black	4304 (9.97)	618 (12.78)	2405 (9.48)	253 (12.01)	1028 (9.48)	
American Indian/ Alaska Native	198 (0.46)	19 (0.39)	126 (0.50)	4 (0.19)	49 (0.45)	
Asian or Pacific Islander	30579 (7.08)	397 (8.21)	1723 (6.79)	163 (7.74)	774 (7.14)	
Sex						0.822
Male	20747 (48.07)	2569 (53.11)	11965 (47.16)	1124 (53.35)	5089 (46.95)	
Female	22409 (51.93)	2268 (46.89)	13407 (52.84)	983 (46.65)	5751 (53.05)	
Histology						0.136
Adenocarcinomas	26065 (60.40)	3397 (70.23)	14918 (58.80)	1428 (67.77)	6322 (58.32)	
Squamous cell carcinoma	13082 (30.31)	1235 (25.53)	7871 (31.02)	564 (26.77)	3412 (31.48)	
Large cell carcinoma	395 (0.92)	76 (1.57)	183 (0.72)	41 (1.95)	95 (0.88)	
Others	3614 (8.37)	129 (2.67)	2400 (9.46)	74 (3.51)	1011 (9.33)	
T-stage						0.722
T1a	11673 (27.05)	486 (10.05)	7709 (30.38)	220 (10.44)	3258 (30.06)	
T1b	8466 (19.62)	749 (15.48)	5171 (20.38)	316 (15.00)	2230 (20.57)	
T2a	17309 (40.11)	2398 (49.58)	9735 (38.37)	981 (46.56)	4195 (38.70)	
T2b	5708 (13.23)	1204 (24.89)	2757 (10.87)	590 (28.00)	1157 (10.67)	
N-stage						0.972
N0	28948 (67.08)	1510 (31.22)	18755 (73.92)	665 (31.56)	8018 (73.97)	
N1	4299 (9.96)	501 (10.36)	2499 (9.85)	219 (10.39)	1080 (9.96)	
N2	8014 (18.57)	2076 (42.92)	3546 (13.98)	889 (42.19)	1503 (13.87)	
N3	1895 (4.39)	750 (15.51)	572 (2.25)	334 (15.85)	239 (2.20)	
Marital status						0.994
Married	23913 (55.41)	2657 (54.93)	14094 (55.55)	1186 (56.29)	5976 (55.13)	
Single	6314 (14.63)	839 (17.35)	3580 (14.11)	330 (15.66)	1565 (14.44)	
Divorced	5503 (12.75)	586 (12.11)	3260 (12.85)	241 (11.44)	1416 (13.06)	
Widowed	7426 (17.21)	755 (15.61)	4438 (17.49)	350 (16.61)	1883 (17.37)	
Tumor size						0.579
1-19	11352 (26.30)	488 (10.09)	7499 (29.56)	222 (10.54)	3143 (28.99)	
20-29	11606 (26.89)	943 (19.50)	7151 (28.18)	392 (18.60)	3120 (28.78)	
>29	20198 (46.80)	3406 (70.42)	10722 (42.26)	1493 (70.86)	4577 (42.22)	
Grade						0.390
Well differentiated; Grade I	7158 (16.59)	281 (5.81)	4727 (18.63)	123 (5.84)	2027 (18.70)	
Moderately differentiated; Grade II	18538 (42.96)	1689 (34.92)	11363 (44.79)	671 (31.85)	4815 (44.42)	
Poorly differentiated; Grade III	16863 (39.07)	2750 (56.85)	8987 (35.42)	1260 (59.80)	3866 (35.66)	
Undifferentiated; Grade IV	597 (1.38)	117 (2.42)	295 (1.16)	53 (2.52)	132 (1.22)	
Laterality						0.812
Left	17882 (41.44)	2075 (42.90)	10454 (41.20)	924 (43.85)	4429 (40.86)	
Right	25274 (58.56)	2762 (57.10)	14918 (58.80)	1183 (56.15)	6411 (59.14)	
Survival statue						0.833
Dead	16714 (38.73)	3794 (78.44)	7916 (31.20)	1630 (77.36)	3374 (31.13)	
Alive	26442 (61.27)	1043 (21.56)	17456 (68.80)	477 (22.64)	7466 (68.87)	

P-value indicates the baseline comparison between training group and test group.

### Independent Risk Factor and Model Construction for Lymph Node Metastasis

**Figure 3:**
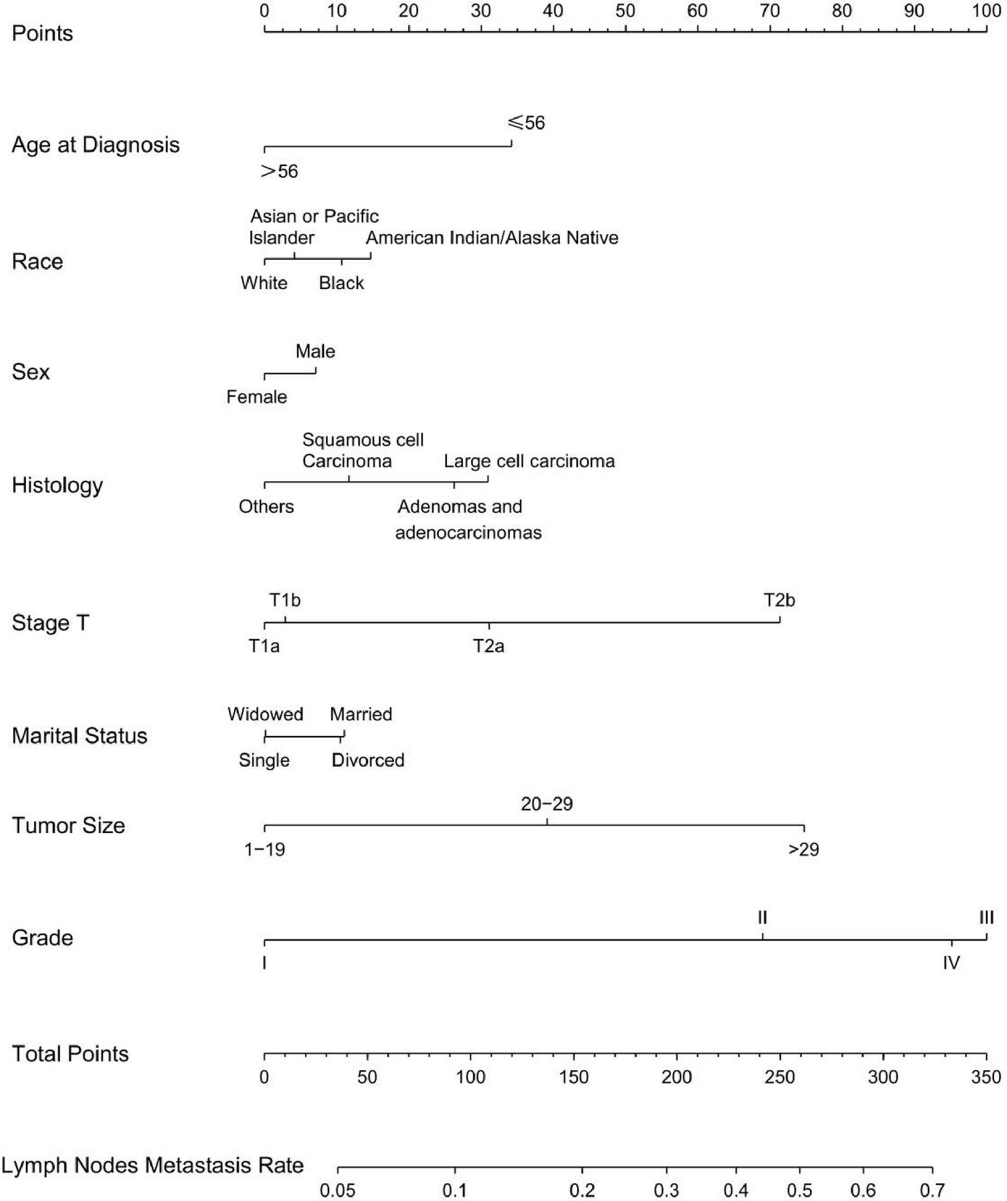
Nomogram for predicting LNM in T1-2N0-3M0 NSCLC. Eight factors were calculated into the LNM prediction nomogram.

**Figure 4:**
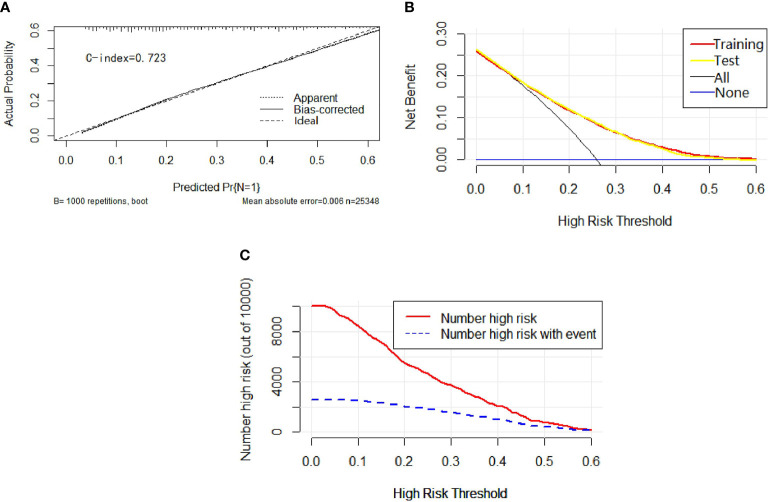
In calibration curve **(A)**, the x-axis represents the predicted probability of LNM, the y-axis refers to the actual probability of LNM, and the ideal line marks the diagonal of the graph, indicating that the predicted probability is utterly equal to the true likelihood, which is the ideal condition of the prediction model. The apparent line represents the theoretical prediction ability, and the bias-corrected stands for the prediction ability of the corrected model. The figure shows that the above three lines are very close, and c-index = 0.723, suggesting that the prediction model is very accurate. In the decision curve **(B)**, the x-axis represents the threshold probability, the y-axis marks the net income, the horizontal blue line is an extreme case that no patients suffer from LNM, and the black line represents another extreme case that all patients have LNM. The red line and the yellow line are predicted by the training group and the test group, respectively. The two lines basically coincide, indicating that the prediction ability of the model is stable and reliable. In the clinical impact curve **(C)**, the x-axis represents the different thresholds of LNM, and the y-axis stands for the number of people. The red line displays the number of high-risk patients under different thresholds, and the blue line illustrates the actual number of LNM in high-risk patients under different thresholds.

Univariable and multivariable binary logistic regression analyses were conducted to screen the independent risk factors for lymph node metastasis. Eight factors, including age, race, sex, histology, T-stage, marital status, tumor size, and grade, were confirmed to work in the prediction of LNM (**Table 3**). Scores assignments and predictive probability for each risk factor in the nomogram (**Figure 3**) were calculated in **Table 5**. The score of each independent predictor is the corresponding upper scale. The total points of each subject are the sum of the scores of each independent predictor. The value of the total points corresponding to the risk axis is the risk of LNM. The higher the total point is, the higher the risk of LNM is. In the training set, the nomogram has good discrimination and calibration in predicting the risk of LNM, and the C index is 0.723 (**Figure 4A**). Decision curve analysis (DCA) of nomogram evaluates the net benefit of patients. The larger the net benefit rate is, the better the predictive performance of the prognostic risk model is (**Figure 4B**). In addition, the clinical impact curve (CIC) detects the predictive value of nomogram in LNM prognosis (**Figure 4C**).

**Table 3:** Logistic regression analysis of the risk factors for lymph node metastasis in cohort N.

Factors	Univariate analysis			Multivariate analysis		
	OR	95%CI	P	OR	95%CI	P
Age						
≤56	Reference			Reference		
>56	0.73	0.67-0.79	<0.001	0.64	0.58-0.70	<0.001
Race						
White	Reference			Reference		
Black	1.25	1.13-1.37	<0.001	1.15	1.04-1.28	0.007
American Indian/ Alaska Native	1.18	0.81-1.69	0.374	1.21	0.81-1.79	0.337
Asian or Pacific Islander	1.05	0.94-1.18	0.354	1.06	0.94-1.19	0.373
Sex						
Male	Reference			Reference		
Female	0.75	0.71-0.79	<0.001	0.91	0.86-0.97	0.004
Histology						
Adenocarcinomas	Reference			Reference		
Squamous cell carcinoma	1.25	1.18-1.33	<0.001	0.82	0.77-0.88	<0.001
Large cell carcinoma	1.95	1.45-2.61	<0.001	1.06	0.76-1.49	0.718
Others	0.53	0.47-0.59	<0.001	0.71	0.62-0.80	<0.001
T-stage						
T1a	Reference			Reference		
T1b	1.85	1.68-2.03	<0.001	1.04	0.91-1.19	0.578
T2a	3.54	3.26-3.83	<0.001	1.50	1.33-1.70	<0.001
T2b	7.37	6.66-8.16	<0.001	2.56	2.20-2.97	<0.001
Marital status						
Married	Reference			Reference		
Single	0.98	0.90-1.06	0.608	0.86	0.79-0.95	0.002
Divorced	1.02	0.93-1.11	0.720	0.99	0.90-1.09	0.882
Widowed	0.86	0.80-0.93	<0.001	0.87	0.79-0.94	0.001
Tumor size						
1-19	Reference			Reference		
20-29	1.93	1.76-2.11	<0.001	1.67	1.48-1.89	<0.001
>29	4.85	4.47-5.25	<0.001	2.67	2.35-3.04	<0.001
Grade						
Well differentiated; Grade I	Reference			Reference		
Moderately differentiated; Grade II	2.89	2.61-3.21	<0.001	2.48	2.22-2.77	<0.001
Poorly differentiated; Grade III	5.09	4.59-5.65	<0.001	3.73	3.34-4.17	<0.001
Undifferentiated; anaplastic; Grade IV	5.10	3.95-6.57	<0.001	3.49	2.62-4.64	<0.001
Laterality						
Left	Reference			Reference		
Right	0.96	0.90-1.01	0.128	0.96	0.91-1.02	0.214

### Independent Risk Factor and Model Construction for Distant Metastasis

**Figure 5:**
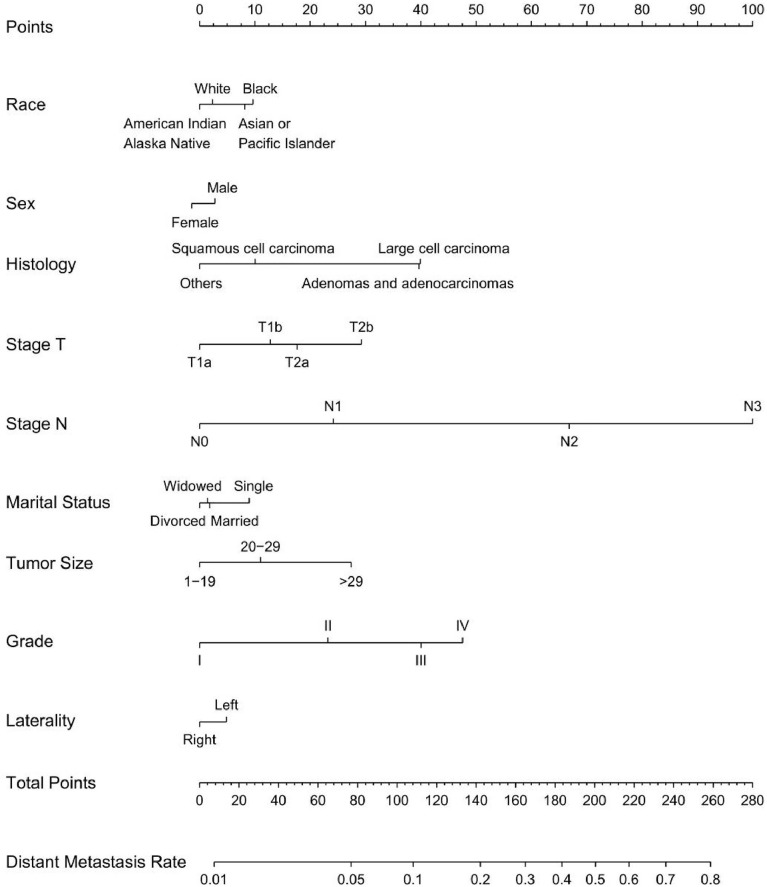
Nomogram for predicting DM in T1-2N0-3M0-1 NSCLC. Nine factors were calculated into DM prediction nomogram.

**Figure 6:**
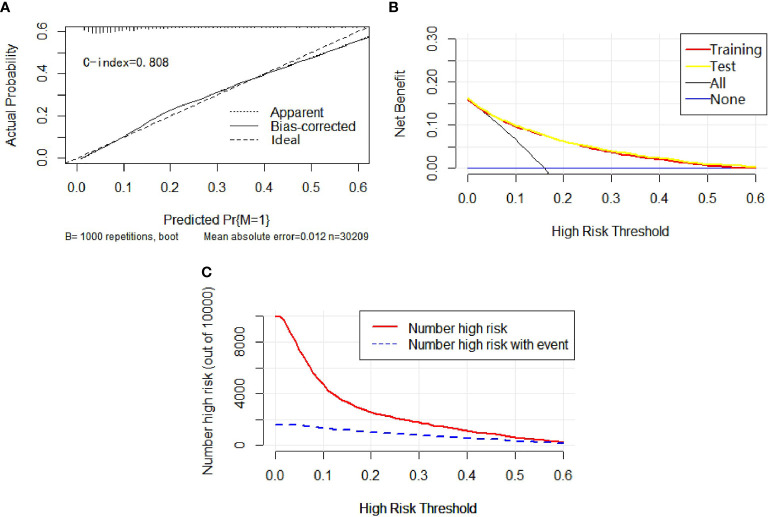
In calibration curve **(A)**, the x-axis represents the predicted probability of DM, the y-axis stands for the actual probability of DM, and the ideal line is the diagonal of the graph, indicating that the predicted probability is utterly equal to the true likelihood, which is the ideal condition of the prediction model. The apparent line represents the theoretical prediction ability, and the bias-corrected marks the prediction ability of the corrected model. The figure reveals that the above three lines are very close, and c-index = 0.808, suggesting that the prediction model is very accurate. In the decision curve **(B)**, the x-axis represents the threshold probability, the y-axis refers to the net income, the horizontal blue line is an extreme case that no patients suffer from DM, and the black line represents another extreme case that all patients have DM. The red line and the yellow line are predicted by the training group and the test group, respectively. The two lines basically coincide, indicating that the prediction ability of the model is stable and reliable. In the clinical impact curve **(C)**, the x-axis represents the different thresholds of DM, and the y-axis marks the number of people. The red line indicates the number of high-risk patients under different thresholds, and the blue line indicates the actual number of DM in high-risk patients under different thresholds.

Univariable and multivariable binary logistic regression analyses were conducted to screen the independent risk factors for distant metastasis. Nine factors, including race, sex, histology, T-stage, N-stage, marital status, tumor size, grade, and laterality, were confirmed to work in the prediction of DM (**Table 4**). Scores assignments and predictive probability for each risk factor in the nomogram (**Figure 5**) are exhibited in **Table 5**. The score of each independent predictor is the corresponding upper scale. The total point of each subject is the sum of the scores of each independent predictor. The value of the total points corresponding to the risk axis is the risk of DM. The higher the total point is, the higher the risk of LNM is. In the training set, the nomogram has good discrimination and calibration in predicting the risk of DM, and the C index is 0.808 (**Figure 6A**). Decision curve analysis (DCA) of nomogram evaluates the net benefit of patients. The larger the net benefit rate is, the better the predictive performance of the prognostic risk model is (**Figure 6B**). Furthermore, the clinical impact curve (CIC) detects the predictive value of nomogram in LNM prognosis (**Figure 6C**).

**Table 4:** Logistic regression analysis of the risk factors for distant metastasis in cohort M.

Factors	Univariate analysis			Multivariate analysis		
	OR	95%CI	P	OR	95%CI	P
Age						
≤56	Reference			Reference		
>56	0.77	0.70-0.84	<0.001	0.92	0.83-1.02	0.108
Race						
White	Reference			Reference		
Black	1.43	1.30-1.57	<0.001	1.18	1.06-1.32	0.002
American Indian/ Alaska Native	0.84	0.50-1.32	0.472	0.95	0.54-1.57	0.846
Asian or Pacific Islander	1.28	1.14-1.43	<0.001	1.15	1.00-1.30	0.039
Sex						
Male	Reference			Reference		
Female	0.79	0.74-0.84	<0.001	0.90	0.84-0.97	0.006
Histology						
Adenocarcinomas	Reference			Reference		
Squamous cell carcinoma	0.69	0.64-0.74	<0.001	0.50	0.46-0.54	<0.001
Large cell carcinoma	1.82	1.38-2.38	<0.001	1.01	0.72-1.39	0.965
Others	0.24	0.20-0.28	<0.001	0.39	0.32-0.47	<0.001
T-stage						
T1a	Reference			Reference		
T1b	2.30	2.04-2.59	<0.001	1.35	1.14-1.61	<0.001
T2a	3.91	3.53-4.33	<0.001	1.52	1.29-1.79	<0.001
T2b	6.93	6.19-7.77	<0.001	2.00	1.67-2.40	<0.001
N-stage						
N0	Reference			Reference		
N1	2.49	2.23-2.78	<0.001	1.77	1.58-1.98	<0.001
N2	7.27	6.74-7.84	<0.001	4.89	4.51-5.30	<0.001
N3	16.29	14.44-18.38	<0.001	10.76	9.49-12.22	<0.001
Marital status						
Married	Reference			Reference		
Single	1.24	1.14-1.35	<0.001	1.17	1.06-1.30	0.002
Divorced	0.95	0.86-1.05	0.3371	0.96	0.86-1.07	0.413
Widowed	0.90	0.83-0.98	0.0216	1.00	0.90-1.11	0.998
Tumor size(mm)						
1-19	Reference			Reference		
20-29	2.03	1.81-2.27	<0.001	1.30	1.10-1.53	0.002
>29	4.88	4.42-5.40	<0.001	1.93	1.64-2.27	<0.001
Grade						
Well differentiated; Grade I	Reference			Reference		
Moderately differentiated; Grade II	2.50	2.20-2.85	<0.001	1.74	1.51-2.00	<0.001
Poorly differentiated; Grade III	5.15	4.54-5.86	<0.001	2.59	2.26-2.98	<0.001
Undifferentiated; anaplastic; Grade IV	6.67	5.21-8.51	<0.001	3.10	2.31-4.13	<0.001
Laterality						
Left	Reference			Reference		
Right	0.93	0.88-0.99	0.028	0.89	0.83-0.95	0.001

**Table 5:** Nomogram score of risk factors for LNM and DM in T1-2 NSCLC.

Risk factors	Nomogram score	
	Lymph nodes metastasis	Distant metastasis
Age		
≤56	34	/
>56	0	/
Race		
White	0	2
Black	11	10
American Indian/ Alaska Native	15	0
Asian or Pacific Islander	4	8
Sex		
Male	7	4
Female	0	0
Histology		
Adenocarcinomas	26	40
Squamous cell carcinoma	12	10
Large cell carcinoma	31	40
Others	0	0
T-stage		
T1a	0	0
T1b	3	13
T2a	31	18
T2b	71	29
N-stage		
N0	/	0
N1	/	24
N2	/	67
N3	/	100
Marital status		
Married	11	2
Single	0	9
Divorced	11	0
Widowed	0	1
Tumor size (mm)		
1-19	0	0
20-29	39	11
>29	75	27
Grade		
Well differentiated; Grade I	0	0
Moderately differentiated; Grade II	69	23
Poorly differentiated; Grade III	100	40
Undifferentiated; anaplastic; Grade IV	95	48
Laterality		
Left	/	5
Right	/	0

### Survival Analyses

**Figure 7:**
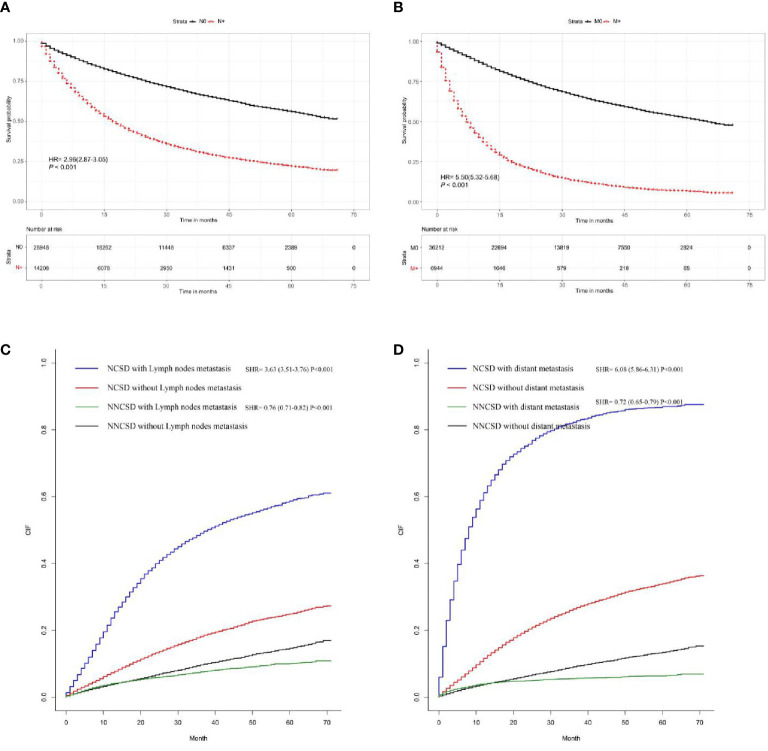
The survival rate of T1-2 NSCLC patients was evaluated by the presence or absence of LNM and DM; the results showed that LNM and DM are closely related to survival rate **(A, B)**. The cancer-specific survival rate of T1-2 NSCLC patients was evaluated by the presence or absence of LNM and DM; the results demonstrated that LNM and DM are closely correlated with cancer-specific survival rates **(C, D)**.

Based on the Kaplan-Meier and Gray method, we analyzed LNM and DM related deaths. The results proved that positive lymph node involvement (hazard ratio (HR) = 2.96, 95%CI = (2.87-3.05), P < 0.001) and distant metastasis (HR = 5.50, 95%CI = (5.32-5.68), P < 0.001) are significantly correlated with overall survival using Kaplan-Meier curves (**Figures 7A, B**). At the same time, the Gray method displayed that LNM (subdistribution hazard ratio (SHR) = 3.63, 95%CI=(3.51-3.76), P < 0.001) and DM (SHR = 6.08, 95%CI = (5.86-6.13), P < 0.001) are significantly correlated with cancer-specific (**Figures 7C, D**).

T1-2 stage NSCLC patients have the following characteristics: the maximum diameter of the primary tumor is ≤7cm, and other tissues in the chest (except visceral pleura) are not involved. Due to different lymph nodes and distant metastasis situations, patients need different diagnosis and treatment methods, and there will be different prognoses. This study showed that about 74% of newly diagnosed T1-2 NSCLC patients are in stage I-IIA without lymph node and distant metastasis. According to NCCN Guidelines(Small Cell Lung Cancer, Version 2020.V6), surgical resection is feasible, and there is no need for radiotherapy or chemotherapy after surgical resection. About 26% of newly diagnosed NSCLC in the T1-2 stage has lymph node metastasis but has no distant metastasis. Some patients (T2bN1M0, T1-2N2-3M0) need postoperative chemoradiotherapy (7). About 16% of newly diagnosed NSCLC in the T1-2 stage has distant metastasis, so there is no indication of radical operation, and other treatments such as chemotherapy, targeted drugs, and immunity are recommended (8). LNM and DM are important factors for making diagnosis and treatment plans and predicting prognosis. At present, the pathological biopsy is still the gold standard for diagnosing LNM and DM in NSCLC. Although there are simple examination methods, PET-CT, LNM and DM can be evaluated preliminarily. Still, the price is higher, and there are false negative and false positive (9). Therefore, non-invasive and effective methods to evaluate the presence of LNM and DM in NSCLC patients are urgently needed. According to the prediction results of the models, further selections of examination and treatment can be more reasonably chose.

In recent years, more and more researches participated in this field, but there are still many shortcomings and limitations. First, previous studies (10, 11) established Cox regression analysis models based on logistic regression analysis. On the contrary, these models cannot be used in clinical practices for their low predictive ability. As a new display form, nomogram can directly display the predicted LNM and DM. This method forms a nomogram to predict the correlation probability, thus providing a reference for further examination and clinical decision-making.

There are a lot of nomograms to predict the diagnosis and prognosis of cancer, but some problems still exist. Some studies include a small sample size (12, 13); some studies contain few factors (14, 15); some studies do not set cut-off value (16, 17); some studies are not divided into training and test groups (18, 19). This is the first and the only study that developed a nomogram to predict the probability of LNM and DM in T1-2 NSCLC patients as far as we know. We divided the included population into the N cohort (T1-2N0-3M0 NSCLC for LNM) and M cohort (T1-2N0-3M0-1 NSCLC for DM). Besides, we divided the LNM cohort and DM cohort into the training group and the test group on the basis of the ratio of 7:3, respectively. The incidence of LNM and DM of T1-2 NSCLC were analyzed, and the corresponding nomograms were constructed through the training group, and then verified by the test group. Two nomograms were established and validated for predicting LNM and DM in patients with T1-2 NSCLC. LNM nomogram includes eight factors, namely age, race, sex, histology, T-stage, marital status, tumor size, and grade, whereas DM nomogram incorporates nine factors, namely race, sex, histology, T-stage, N-stage, marital status, tumor size, grade, and laterality. Both of the nomograms indicated good agreement between predictions and observations. C-index of the LNM nomogram, and DM nomogram were calculated with the values of 0.723 and 0.808, respectively. These nomograms reveal good clinical utility in the proper threshold probability range.

In this population-based study, from the score table of nomogram (**Table 5**), it is obvious that grade, tumor size, and T-stage account for the most significant score. It has been reported that grade is closely related to LNM in NSCLC (20). Our data also showed that the LNM risk of moderately differentiated, poorly differentiated and undifferentiated cancer increased to 2.48, 3.73 and 3.49 compared with well-differentiated carcinoma (both P < 0.001). In this study, patients with squamous cell carcinoma have lower risk in comparison with adenocarcinomas patients. Previous studies (21) have revealed that young age at diagnosis is associated with the increased risk of LNM in patients with NSCLC. As in these studies, we noticed that the risk of LNM was higher in the younger T1-2 NSCLC group (age ≤ 56 years) than that in the older T1-2 NSCLC group (age > 56 years). The reason may be that young patients with lower tumor differentiation grade are more likely to escape from the immune surveillance of the body. But for this conjecture, we still have not come to any conclusive data, which needs further study. Adenocarcinomas are more likely to have lymph node metastasis than squamous cell carcinoma (P < 0.001). No significant differences in LNM between left and right lung cancer were found.

For the DM nomogram, the largest proportion in risk scores factors are T-stage, Grade, and Histology. Of note, patients with the worse N-stage and worse grade are more prone to occur distant metastasis. As are consistent with previous studies (22), adenocarcinomas are more likely to have distant metastasis than squamous cell carcinoma, and female are more prone to have distant metastasis than male. Different from the predicted LNM, there were no significant differences between the older group (> 56) and the younger group (≤56). Laterality has been found to be predictive of distant metastasis in T1-2 NSCLC. Compared with left lung cancer, right lung cancer has a lower risk of distant metastasis(HR=0.89, 95%CI=0.83-0.95, P<0.001). The reason may be related to the anatomical structure of the lung. For instance, the left lung is divided into 2 pages and 8 lung segments, and the right lung is divided into 3 pages and 10 lung segments. The right pulmonary artery is longer, while the left pulmonary artery is shorter.

Furthermore, we found that LNM and DM in T1-2 NSCLC were associated with cancer-specific death and non-cancer-specific death. In this database-based study, we screened 43,156 eligible patients with a median follow-up of 70 months from real-world data. We analyzed the data by adopting appropriate statistical methods and found these convincing conclusions.

However, there are some limitations in this study. Firstly, this is a population-based retrospective analysis that lacks prospective data for verification. Although the 8th edition of TNM is currently used, we can only continue to refer to the 7th edition (**Table 6**) because the study is a retrospective study and the seventh edition was used for case entry at that time. Secondly, this database’ information is insufficient in terms of smoking, tumor markers, imaging examination, and important molecular factors (EGFR, rose1 and ALK gene status), thus leading to that our nomogram failed to include these important factors. Finally, our data are only from one institution. Although the data are divided into training and test groups, it inevitably causes internal bias. We can further collect multi center data and combine other factors for the improvement of the model.

**Table 6:** 7th Edition AJCC Cancer Staging Manual for NSCLC.

T component	
0 cm (pure lepidic adenocarcinoma ≤3 cm in total size)	T1a if ≤ 2 cm; T1b f>2-3cm
≤0.5 cm invasive size (lepidic predominant adenocarcinoma ≤ 3 cm total size)	T1a if ≤ 2cm; T1b if>2-3cm
≤1cm	T1a
>1-2 cm	T1a
>2-3 cm	T1b
>3-4 cm	T2a
>4-5 cm	T2a
>5-7 cm	T2b
>7 cm	T3
Bronchus <2 cm from carin	T3
Total atelectasis/pneumonitis	T3
Invasion of diaphragm	T3
Invasion of mediastinal pleura	T3
**N component**	
Regional lymph nodes cannot be assessed	NX
No regional lymph node metastasis	N0
Metastasis in ipsilateral peribronchial and/or ipsilateral hilar lymph nodes andintrapulmonary nodes, including involvement by direct extension	N1
Metastasis in ipsilateral mediastinal and/or subcarinal lymph node(s)	N2
Metastasis in contralateral mediastinal, contralateral hilar, ipsilateral or contralateral scalene, or supraclavicular lymph node(s)contralateral scalene, or supraclavicular lymph node(s)	N3
**M component**	
Metastasis within the thoracic cavity	M1a
Single extrathoracic metastasis	M1b
Multiple extrathoracic metastasis	M1b

In conclusion, based on the independent risk factors screened from the large database, we constructed two nomograms that can accurately predict LNM and DM in different stages of T1-2 NSCLC patients. The listed factors can be easily obtained from clinical and pathological data. Through the verification of discrimination and correction, our nomogram has high accuracy and reliability, so it can be applied to clinical practices. Combined with other clinical data, it can help doctors make better diagnosis and investigation, individualized treatment, and follow-up management decisions for t1-2 NSCLC patients.

## Data Availability Statement

The datasets presented in this study can be found in online repositories. The names of the repository/repositories and accession number(s) can be found in the article/**Supplementary Material**.

## Author Contributions

YQ, SW, and JL designed the study. WY, LZ, and YS extracted and analyzed the data. BZ and LT wrote and edited the manuscript. Authors were ranked according to their contributions. YQ and SW contributed equally to this work and should be considered as co-first authors. All authors contributed to the article and approved the submitted version.

## Funding

National Natural Science Foundation of China (81673809); Science and technology project of traditional Chinese medicine in Zhejiang Province (2020ZB049); Project of Zhejiang lung cancer prevention and treatment center of traditional Chinese medicine (2A11801); Zhejiang Provincial natural science foundation (LQ19H030004); Key research department project of Oncology Department of Tongde Hospital of Zhejiang Province.

## Conflict of Interest

The authors declare that the research was conducted in the absence of any commercial or financial relationships that could be construed as a potential conflict of interest.

## Publisher’s Note

All claims expressed in this article are solely those of the authors and do not necessarily represent those of their affiliated organizations, or those of the publisher, the editors and the reviewers. Any product that may be evaluated in this article, or claim that may be made by its manufacturer, is not guaranteed or endorsed by the publisher.
