# Supplementary material for: Development of Nomograms for Predicting Lymph Node Metastasis and Distant Metastasis in Newly Diagnosed T1-2 Non-Small Cell Lung Cancer: A Population-Based Analysis
Source: Front Oncol. 2021 Sep 8;11:683282. doi: 10.3389/fonc.2021.683282 (PMC8456089; doi:10.3389/fonc.2021.683282)
Supplement: Supplementary file 1 [file DataSheet_1.pdf]

Comparison of the Descriptors in the Eighth Edition of the TNM Classification of Lung Cancer Compared with the Seventh Edition*

**Descriptor**	**7th Edition T/N/M**	**8th Edition T/N/M**
**T component**		
0 cm (pure lepidic adenocarcinoma ≤3 cm in total size)	T1a if≤2 cm; T1b f>2-3cm	Tis (AIS)
≤0.5 cm invasive size (lepidic predominant adenocarcinoma≤3 cm total size)	T1a if≤2cm; T1b if>2-3cm	T1mi
≤1cm	T1a	T1a
>1-2 cm	T1a	T1b
>2-3 cm	T1b	T1c
>3-4 cm	T2a	T2a
>4-5 cm	T2a	T2b
>5-7 cm	T2b	T3
>7 cm	T3	T4
Bronchus <2 cm from carina	T3	T2
Total atelectasis/pneumonitis	T3	T2
Invasion of diaphragm	T3	T4
Invasion of mediastinal pleura	T3	—
**N component**		
No assessment, no involvement, or involvement of regional lymph nodes	NX, NO, N1, N2, N3	No change
**M component**		
Metastasis within the thoracic cavity	M1a	M1a
Single extrathoracic metastasis	M1b	M1b
Multiple extrathoracic metastasis	M1b	M1c
